# Effects of Self-ligating Orthodontic Appliances on the Periodontal Health of Adolescents: A Prospective Cohort Study

**DOI:** 10.3290/j.ohpd.b965741

**Published:** 2021-02-19

**Authors:** Aikaterini Papadimitriou, Georgios Kouvelis, Theodora Fanaropoulou, Ioannis Doulis, Merakou Kyriakoula, Anastasia Mparmpouni, Dimitrios Kloukos

**Affiliations:** a Researcher, Department of Orthodontics and Dentofacial Orthopedics, 251 Hellenic Airforce V.A. General Hospital, Athens, Greece. Performed all clinical assessments and measurements, wrote the manuscript, prepared all figures, reviewed the manuscript.; b Researcher, Department of Orthodontics and Dentofacial Orthopedics, 251 Hellenic Airforce V.A. General Hospital, Athens, Greece. Assisted with interpretation of statistics, reviewed the manuscript.; c Professor, Department of Public and Administrative Health, National School Of Public Health, Athens, Greece. Supervised the project, assisted with writing the manuscript, reviewed the manuscript.; d Researcher, Department of Orthodontics and Dentofacial Orthopedics, 251 Hellenic Airforce V.A. General Hospital, Athens, Greece; Department of Orthodontics and Dentofacial Orthopedics, School of Dental Medicine, University of Bern, Bern, Switzerland. Performed all clinical assessments and measurements, wrote the manuscript, prepared all figures, supervised the project, assisted with writing the manuscript, reviewed the manuscript.

**Keywords:** fixed orthodontic appliances, gingival index, periodontal health, plaque index

## Abstract

**Purpose:**

To evaluate the association between orthodontic treatment with fixed appliances and periodontal health during treatment by examining gingival inflammation indices and saliva properties.

**Materials and Methods:**

Thirty consecutive orthodontic patients, aged 11–18 years old, who were eligible for fixed orthodontic appliances, were included in the study. Plaque index (PI), gingival index (GI), salivary pH and flow rate were recorded at three timepoints: immediately before placement of orthodontic fixed appliances (T_0_), and 1 (T_1_) and 3 months (T_2_) after bonding.

**Results:**

The hypothesis that PI would remain constant across timepoints was rejected. PI increased over time (0 to 1 scale, T_1_-T_0_: mean diff. = 0.10, 95% CI = 0.03, 0.18, p = 0.01; T_2_-T_0_: mean diff. = 0.16, 95% CI = 0.08, 0.24, p < 0.001). On the other hand, GI changed statistically significantly over time (p = 0.05). Patients’ age was not a predictor for PI change (p = 0.93), but it was for GI (p = 0.01). As anticipated, average PI was found to be higher for the mandibular dentition by 0.10 (95% CI = 0.04, 0.16) and the labial surfaces of teeth of both jaws by 0.51 (95% CI = 0.45, 0.57).

**Conclusions:**

Within the framework of the current study, orthodontic treatment appeared to affect the periodontal health of patients, but the changes were clinically negligible and not consistently statistically significant.

Although orthodontic fixed appliances are an integral part of contemporary treatment planning, they can at the same time be considered a serious risk factor for enamel integrity and/or periodontal health; this is mainly attributed to plaque accumulation and colonisation by unfavourable oral microbes.^40^ Moreover, fixed orthodontic appliances may impede oral hygiene procedures and alter the oral microflora by reducing the pH and promoting plaque development.^1^ This may, in turn, lead to an increased risk for developing gingivitis, white spot lesions and halitosis.^25,38^

The age of orthodontic patients usually lies in the period of adolescence, a transitional period which may give rise to difficulties for patients, their parents/guardians and the orthodontist.^1^ Dietary habits, parents’ role, social status and patient education are also important where oral health status or promotion is concerned. It is therefore necessary for orthodontists to consider several factors when advising patients on oral health.^17,21,28^

In a recent study by Kudirkaite et al,^18^ 16- to 18-year-old orthodontic patients appeared to have better oral health than those who were 12–15 years of age during their treatment. Females more effectively performed oral hygiene measures than did males.^36^ Another study^17^ confirmed the same results. Gong et al^15^ reported that orthodontic patients had higher plaque and bleeding indices in the first six months of treatment with fixed appliances, and that girls were more consistent in toothbrushing than boys. Other recent studies^24,34^ found elevated plaque and bleeding in orthodontic patients, with girls aged 15-18 showing the lowest percentage of these markers. The study by Andjelić et al^2^ demonstrated a strong correlation between orthodontic treatment and plaque and bleeding indices. Finally, Babacan et al^6^ found that plaque and bleeding markers were increased only during the 1st and 4th week of orthodontic treatment, whereas the results of the study by Ghijselings et al^14^ suggested an overall increase of the indices throughout orthodontic treatment up to completion.

Saliva possesses the ability to influence oral hygiene; alterations in salivary pH and flow rate play a significant role in proper oral function and the appearance of caries.^13,19^ Treatment with Invisalign, as reported by Schaefer et al,^33^ did not alter simulated saliva flow rate; measurements remained constant during the first 8 months of treatment. Conversely, in patients with fixed appliances, salivary flow rate has been shown to increase, as reported in previous studies.^12,22^

Salivary pH demonstrated a significant increase in the 1st month of treatment with fixed appliances,^22^ as opposed to other studies which reported pH level alterations after 3 months of orthodontic treatment.^12^ A possible explanation for this may be that orthodontic appliances increase the retentive plaque surfaces on teeth, causing elevated amounts of hydrogen ions in oral environment, which, in turn, decrease pH.^20^ Thus, it would be beneficial to observe the possible changes of these two parameters also in orthodontic patients treated with self-ligating brackets.

The primary objective of this study was to identify the effect of orthodontic treatment on the periodontal health of adolescent patients with self-ligating brackets after placement of appliances. The secondary objective was to detect possible alterations in salivary flow rate and pH.

## Materials and Methods

### Study Sample

The sample for this study was recruited from patients presenting for treatment at the Orthodontic Department of the 251 Air Force General Hospital, Athens, Greece between March and October 2017. The following eligibility criteria were used to select appropriate patients for this study: adolescents (12–18 years of age) of any sex, no reported oral habits affecting periodontal health, including smoking, systemic diseases, or any medication affecting the oral cavity taken within the last 3 months, no teeth with active caries and/or missing teeth due to caries, absence of previous or active periodontal disease.

The patients’ orthodontic treatment plan did not include tooth extractions or other mechanics requiring the use of bands on molars. Ethics board approval was obtained before study commencement (approval number: 076/6271/ 1404, 2017) and written informed consent was obtained from all patients or their guardians.

### Sample Size Calculation

Sample size was calculated using the “guided study design” mode of GLIMMPSE (http://glimmpse.samplesizeshop.org/) for repeated-measures studies. Statistical power was set to 0.8, repeated measures were set to 0, 30, and 90 days. The primary hypothesis was set to treatment-by-time interaction, the statistical test employed was Hotelling-Lawley Trace test, and type I error was set to 0.05. Finally, correlations were set to LEAR (linear exponent firstorder autoregressive). With these assumptions, a sample size of 30 participants in total was acquired.

### Clinical Procedures

Study participants received fixed orthodontic treatment with self-ligating brackets and nickel-titanium (NiTi) archwires in both arches (metallic labial brackets/tubes, Innovation-R and Sentalloy Wire 0.014-inch, both from GAC International; Central Islip, NY, USA) for three months.

Three weeks before beginning orthodontic treatment, each patient received professional oral care and standardised hygiene instructions using a typodont model, with specific attention to fixed appliance care. The bonding procedure was performed with the direct technique using Transbond-XT (3M Oral Care; St Paul, MN, USA). All patients were asked to refrain from eating, drinking, and brushing 2 h prior to all clinical examinations and saliva collection. All procedures were performed between 09:00 and 12:00 am. After completion of orthodontic bonding, all patients again received the same oral hygiene instructions.

### Salivary Flow Rate

In order to estimate salivary flow rate, patients were asked to chew a paraffin pellet for 5 min. The patient was instructed not to swallow any quantity of the saliva, which was collected in sterile urine boxes. After 5 min of stimulated salivation, saliva foams were removed, and the procedure was completed by measuring the remaining saliva with one-usage syringes. All saliva measurements that were performed in the clinic were carried out approximately between 09:00 and 11:00 am, because it has been shown that the amount of saliva secretion varies during the day.^37^

### pH Levels

The pH levels of all saliva samples were estimated using pH-indicator strips (Neutralist, Merck; Darmstadt, Germany). Using this technique, pH values were determined with an accuracy of 0.5 and a range of 5.0 to 10.0 on the pH scale.

### Periodontal Indices

PI and GI were taken using a Red-Cote Liquid Disclosing Solution (Sunstar Americas; Schaumburg, IL, USA) and a standard periodontal probe (PB) (CPU 15 UNC, Hu-Friedy; Chicago, IL, USA), respectively, and subsequently recorded in customised forms. Measurements were carried out on all teeth mid-facially, distally and mesially on the buccal and lingual/palatal aspects of each tooth. Plaque was then scored in each area based on the original Silness and Löe Plaque Index^35^ with the application of the Red-Cote Liquid Disclosing Solution on all surfaces. For both indices, possible scores were 0 for absence or 1 for presence of plaque or bleeding in each measurement. Thus, the mean score ranged from 0 to 1 in each patient, taken from 168 measured surfaces of 28 teeth.

As for the GI measurements, the same periodontal probe was placed along the tooth axis into the sulcus of each measured surface, with the same numbers as PI for each surface and using the same scoring system as PI for each surface and patient.

All measurements were performed by the same clinician (AP) after calibration under the guidance of a periodontal specialist on 10 patients who were not eligible for inclusion in the study. Intra-examiner calibration was repeated a week later in the same patients, but no statistical correlation was calculated due to the dynamic nature of the outcome.

### Statistical Analysis

The primary null hypothesis was that the mean of each of the periodontal indices, PI and GI, remained stable across the three measurements. The primary null hypothesis was tested by fitting linear mixed models. Bonferroni correction to counteract type I error inflation was performed. The pairwise comparisons (T_0_-T_1_, T_1_-T_2_, T_0_-T_2_) were assessed using the models described above. Subsequently, age, side of tooth (labial or palatal/lingual), jaw (maxilla or mandible) and tooth group (maxillary anterior, mandibular anterior, premolars, molars) were included one at a time in the fixed portion of the mixed models, both as main effect and as interaction with time in order to test their effect on PI and GI.

Model selection was conducted with likelihood-ratio tests between each of the above models and the respective nested ones without the interaction term. PI and GI at T_0_ and T_2_, respectively were tested for association with Kruskal-Wallis tests. Statistical significance was set to α = 0.05. All statistical analyses and graphical plots were conducted using Stata 13.0/SE software (StataCorp; College Station, TX, USA).

## Results

The study sample consisted of 30 consecutive adolescent patients (13 girls, 17 boys, mean age 13.97 ± 2.07 years) satisfying the inclusion criteria. No loss to follow-up was recorded at any time point.

### Clinical Results (Repeated Measurements of Periodontal Indices)

Corresponding PI comparisons between pairs of time are shown in Table 1. The basic assumption that PI was stable over time during study follow-up was rejected (p < 0.001). There was marginal statistical significance that GI changed over time (p = 0.05).

**Table 1:** The pairwise comparisons from mixed models with PI and GI as the dependent variables and time as the explanatory variable

	Mean (SE)	95% CI	p-value
**PI**			
T_1_-T_0_	0.10 (0.04)	(0.02, 0.18)	0.01
T_2_-T_0_	0.16 (0.04)	(0.08, 0.24)	<0.001
T_2_-T_1_	0.06 (0.01)	(-0.02, 0.14)	0.13
**GI**			
T_1_-T_0_	0.01 (0.04)	(-0.08, 0.08)	0.91
T_2_-T_0_	-0.08 (0.04)	(-0.16, 0.01)	0.05
T_2_-T_1_	-0.08 (0.04)	(-0.16, -0.01)	0.04

PI: plaque index, GI: gingival index, SE: standard error, CI: confidence interval.

Pair comparisons (Table 1) particularly showed how PI increased over time: The mean difference between T_1_ and T_0_ was estimated to be 0.10 (p = 0.01, 95% CI = 0.02, 0.18), and the mean difference between T_2_ and T_0_ was estimated to be 0.16 (< 0.001, 95% CI = 0.08, 0.24). The corresponding changes in GI were not statistically significant for T_1_-T_0_ (mean diff. = 0.01, p = 0.91, 95% CI = -0.08, 0.08) and slightly significant for T_2_-T_0_ (mean difference = -0.08, p = 0.05, 95% CI = -0.16, -0.01). Overall, PI changed in patients over time, increasing at one (T_1_) and three months (T_2_) after bonding (T_0_), but no statistically significant difference was recorded between T_1_ and T_2_. GI slightly decreased over time, but statistical significance was not found between measurements at all timepoints (T_1_-T_0_: p = 0.91; T_2_-T_0_: p = 0.05; T_2_-T_1_: p = 0.04).

Factors that may have interacted with PI and GI changes were documented: age of the patients, buccal surfaces vs palatal-lingual surfaces, maxilla vs mandible, as well as the various groups of teeth (maxillary anterior, mandibular anterior, premolars, molars).

The interaction tests for the PI models showed no statistically significant interaction for any model. The corresponding tests for GI showed that only the jaw as variable (maxilla vs mandible) yielded statistically significant results (p = 0.04) (Table 2), with the mandible presenting higher GI.

**Table 2:** The results of the likelihood-ratio tests for the interaction terms with PI and GI as the dependent variables and patient’s age, jaw and surface as main effects in the fixed portion of models

	X^2^ statistic	p-value
**PI**		
age	3.63	0.16
surface (labial vs palatal)	0.10	0.95
jaw (maxilla vs mandible)	0.07	0.97
**GI**		
age	4.35	0.11
surface (labial vs palatal)	1.13	0.57
jaw (maxilla vs mandible)	6.32	0.04

PI: plaque index, GI: gingival index.

The results of the mixed models with the labial PI and GI as dependent variables and the group of teeth are reported in Table 3. The PI of the different tooth groups appeared to change differentially over time. PI increased significantly from T_0_ to T_1_ (p = 0.01) for maxillary anterior teeth. This increase was estimated to be 0.15 (95% CI = 0.05, 0.25). The corresponding increase between T_2_-T_0_ was also statistically significant (p-value < 0.001) and was estimated to be 0.24 (95% CI = 0.14, 0.34). Coompared to the maxillary anterior teeth, the molars appear to have a higher PI by 0.16 (95% CI = 0.06, 0.26) for the period T_2_-T_0_. For the modified GI, no statistically significant result was observed over time for any tooth group (Table 3).

**Table 3:** The results of the mixed models with modified PI and GI as dependent variables respectively and groups of teeth included in the fixed part of the models both as main effects and as interaction with time

	Estimate (SE)	95% CI	p-value
**Modified PI (only labial surfaces)**			
upper anterior at T_0_ (reference)	0.56 (0.04)	(0.48, 0.64)	<0.001
main effects			
T_1_	0.15 (0.05)	(0.05, 0.25)	0.01
T_2_	0.24 (0.05)	(0.14, 0.34)	<0.001
lower anterior (T_2_-T_0_)	0.01 (0.05)	(-0.09, 0.11)	0.80
premolars (T_2_-T_0_)	-0.04 (0.05)	(-0.14, 0.06)	0.44
molars (T_2_-T_0_)	0.16 (0.05)	(0.06, 0.26)	0.01
**Modified GI (only labial surfaces)**			
upper anterior at T_0_ (reference)	0.24 (0.04)	(0.16, 0.32)	<0.001
Main effects			
T_1_	-0.01 (0.05)	(-0.11, 0.09)	0.84
T_2_	-0.09 (0.05)	(-0.18, 0.01)	0.07
lower anterior (T_2_-T_0_)	-0.07 (0.05)	(-0.17, 0.02)	0.13
premolars (T_2_-T_0_)	-0.04 (0.05)	(-0.13, 0.06)	0.46
molars (T_2_-T_0_)	0.02 (0.05)	(-0.08, 0.12)	0.68

PI: plaque index, GI: gingival index, SE: standard error, CI: confidence interval.

### Salivary Flow Rate and pH

**Fig 1:**
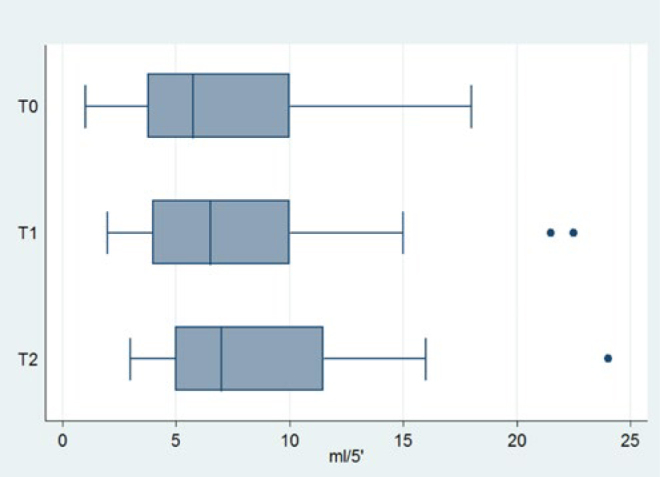
Box plots of salivary flow rate.

**Fig 2:**
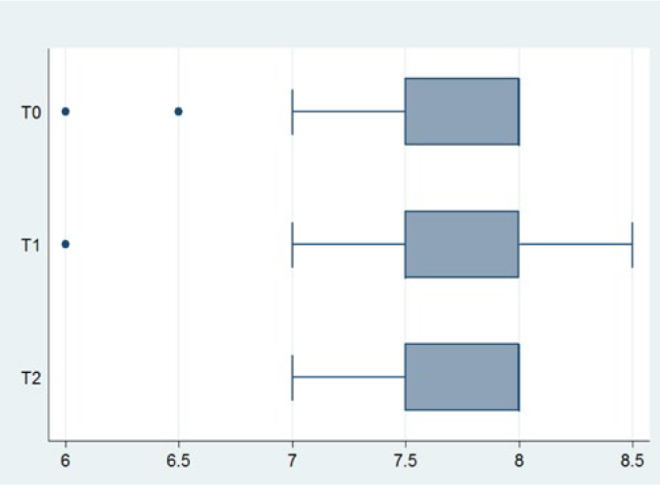
Box plots of salivary pH.

Descriptive statistics for saliva flow rate and pH at each timepoint are reported in Table 4. Mean flow rate and pH appeared to increase with time. Box-plots of saliva flow rate and pH at each timepoint are illustrated in Figs 1 and 2, respectively. Salivary properties remained essentially unaltered throughout the entire study follow-up.

**Table 4:** Descriptive statistics for flow rate and pH, for T_0_, T_1_ and T_2_

	Variable	Mean	sd	min	max	Q1	Median	Q3
TO	Flow rate (ml/5 min)	7.08	4.33	1	18	3.8	5.75	10
	pH	7.63	0.51	6	8	7.5	8	8
								
T1	Flow rate (ml/5 min)	7.93	5.15	2	22.5	4	6.5	10
	pH	7.67	0.46	6	8.5	7.5	7.5	8
								
T2	Flow rate (ml/5 min)	8.35	4.83	3	24	5	7	11.5
	pH	7.78	0.28	7	8	7.5	8	8

sd: standard deviation, Q1: quartile 1, Q3: quartile 3.

## Discussion

Despite the technical progress and common use, orthodontic treatment with fixed appliances often impairs adequate oral hygiene, providing niches for food residue and bacteria.^20,28,38^ Unfavourable effects on both dental hard tissue^4,32^ and the periodontium^27,32^ owing to orthodontic bands and brackets have been documented; several investigations reported that the most important factor in the development of periodontal diseases may be the presence of plaque at the gingival margin.^4,40^ The pertinent literature indicates that adolescent patients tend to accumulate more plaque than adults,^8,9^ while the influence of gender seems rather limited.^36^

It is therefore important that patients maintain proper oral health and use additional oral hygiene measures during the course of orthodontic therapy.^15,30^

The primary objective of this study was to illustrate the effect of fixed orthodontic treatment on the oral health of adolescent patients with self-ligating orthodontic appliances in the first trimester of treatment. According to previous studies, the accumulation of microbial plaque increases after commencement of orthodontic treatment and it is difficult to remove from braces.^15,30^ Most changes were found to occur at the beginning of treatment, due to alteration in the oral environment. For instance, the patients with fixed appliances must learn how to brush and maintain good oral hygiene in this new situation in the first month of treatment. As far as bracket type is concerned, two recent systematic reviews reported no significant difference between self-ligating and conventional braces when the oral health of orthodontic patients was evaluated.^5,39^ In contrast, other studies reported the advantage of self-ligating brackets in terms of oral hygiene and avoidance of decalcification of teeth, due to cross-contamination of elastomeric modules.^28^

In the present study, periodontal indices were assessed at three timepoints: exactly before start of treatment (T_0_), one month (T_1_), and three months later (T_2_). These indices were then explored for their correlation with possible influencing factors: age of patients, maxilla vs mandible, different surfaces of the teeth and different groups of teeth, which were partly investigated in previous research.^1,2,14, 15,18,24,28,34,38,40^

The null hypothesis, that PI would remain stable over time, was rejected; PI actually increased over time. On the contrary, GI did not change statistically significantly over time. This is in line with previous studies that also reported no statistically significant differences of GI between the measurements.^2,15,18,24,34^ It is interesting to note that GI scores at 4–6 weeks had slightly decreased in two studies.^11,16^ This may be attributed to the observation period chosen, which could be regarded as too short to allow the establishment of any real biological change, and which might only reflect the result of patients’ initially high ambitions in oral hygiene.

Patient age, as a prognostic factor for oral health maintenance, was not statistically significant for either PI or GI changes. Previous studies found that the group of 16- to 18-year-old patients presented better oral hygiene.^17,18,26^

In terms of the maxilla or mandible, only GI was statistically significant and increased in T_1_ and T_2_. As for the different tooth groups and how they are affected, PI appeared to change differentially over time. PI increased statistically significantly from T_0_ to T_1_ for maxillary anterior teeth. The corresponding increase between T_2_-T_0_ was also statistically significant. With reference to the maxillary anterior teeth, the molars appeared to have a higher PI by 0.16 for the period T_2_-T_0_. For the modified GI, no statistically significant result was observed over time for any tooth group.

Salivary flow rate is regarded as an essential physical parameter for oral health conservation; its protective contribution has been documented.^13^ Stimulated salivary flow rate of less than 0.2 ml/min is an abnormal situation,^19^ which could be a potential risk factor for caries. In orthodontic patients with fixed appliances, Bonetti et al^7^ found no difference after 1 year of active orthodontic treatment. In the short term, Lombardo et al^23^ found that after 4 and 8 weeks of active fixed orthodontic treatment, salivary flow rate remained unaltered; Arab et al^3^ found the same after 6, 12, 18 weeks with fixed appliances. On the other hand, other studies^17,38,41,58^ found a statistically significant increase after 1 month of fixed orthodontic treatment.^10,20,22,31^ Peros et al^29^ found a statistically significant increase after 18 weeks of fixed orthodontic treatment. According to our findings, the null hypotheses that salivary flow rate does not change from T_0_ to T_2_ was rejected (p = 0.006). Salivary flow rate increased over time after the placement of fixed orthodontic appliances. Nevertheless, the noted difference may be regarded as clinically unimportant.

Low salivary pH has been associated not only with the presence of caries^12^ but also with periodontal disease.^22^ When fixed orthodontic appliances are concerned, conflicting outcomes have been reported. Li et al^22^ found no difference in pH levels after 1, 3 and 6 months of active fixed orthodontic treatment, as did Bonetti et al^7^ after 1 year. Lara-Carillo et al^20^ found a statistically significant increase after 1 month of fixed orthodontic treatment, while Arab et al^3^ found that after 6, 12 and 18 weeks with fixed appliances, pH statistically significantly decreased. Our results indicated that there is a slight increase over time, but showed no statistically significant change in pH at the initial stages of fixed orthodontic treatment.

As a final remark, it should be noted that periodontal health is influenced by several factors. Malocclusion and, subsequently, orthodontic treatment may have only a limited effect compared to behavioural influences (smoking, oral hygiene, habits, diet), along with genetic background. Effective brushing should be the primary concern of young patients while undergoing orthodontic treatment to maintain high levels of oral hygiene, irrespective of the bracket system used.

## Conclusions

In the current study, orthodontic treatment appeared to affect the periodontal health of patients, but clinical aspects were negligible. There was an increase of PI over time, and at the same time, GI changed marginally statistically significantly. Salivary properties remained essentially unaltered throughout the follow-up period.

## References

[ref1] Ahn SJ, Lee SJ, Lim BS, Nahm DS (2007). Quantitative determination of adhesion patterns of cariogenic streptococci to various orthodontic brackets. Am J Orthod Dentofacial Orthop.

[ref2] Andjelić J, Matijević S (2014). Condition of periodontium in patients with fixed orthodontic appliances. Vojnosanit Pregl..

[ref3] Arab S, Nouhzadeh Malekshah S, Abouei Mehrizi E, Ebrahimi Khanghah A, Naseh R, Imani MM (2016). Effect of fixed orthodontic treatment on salivary flow, pH and microbial count. J Dent (Tehran).

[ref4] Arends J, Christoffersen J (1986). The nature of early caries lesions in enamel. J Dent Res.

[ref5] Arnold S, Koletsi D, Patcas R, Eliades T (2016). The effect of bracket ligation on the periodontal status of adolescents undergoing orthodontic treatment. A systematic review and meta-analysis. J Dent.

[ref6] Babacan H, Sokucu O, Marakoglu I, Ozdemir H, Nalcaci R (2011). Effect of fixed appliances on oral malodor. Am J Orthod Dentofacial Orthop.

[ref7] Bonetti GA, Parenti SI, Garulli G, Gatto MR, Checchi, L (2013). Effect of fixed orthodontic appliances on salivary properties. Prog Orthod.

[ref8] Boyd RL, Baumrind S (1992). Periodontal considerations in the use of bonds or bands on molars in adolescents and adults. Angle Orthod.

[ref9] Boyd RL, Leggott PJ, Quinn RS, Eakle WS, Chambers D (1989). Periodontal implications of orthodontic treatment in adults with reduced or normal periodontal tissues versus those of adolescents. Am J Orthod Dent Orthop.

[ref10] Cardoso AA, Lopes LM, Rodrigues LP, Teixeira JJ, Steiner-Oliveira C, NobreDos-Santos M (2017). Influence of salivary parameters in the caries development in orthodontic patients-an observational clinical study. Int J Paediatr Dent.

[ref11] Cardoso Mde A, Saraiva PP, Maltagliati LA, Rhoden FK, Costa CC, Normando D, Capelozza Filho L (2015). Alterations in plaque accumulation and gingival inflammation promoted by treatment with self-ligating and conventional orthodontic brackets. Dental Press J Orthod.

[ref12] Chang HS, Walsh LJ, Freer TJ (1999). The effect of orthodontic treatment on salivary flow, pH, buffer capacity and levels of mutans streptococci and lactobacilli. Aust Orthod J.

[ref13] Dawes C (2003). Estimates, from salivary analyses, of the turnover time of the oral mucosal epithelium in humans and the number of bacteria in an edentulous mouth. Arch Oral Biol.

[ref14] Ghijselings E, Coucke W, Verdonck A, Teughels W, Quirynen M, Pauwels M, Carels C, van Gastel J (2014). Long-term changes in microbiology and clinical periodontal variables after completion of fixed orthodontic appliances. Orthod Craniofac Res.

[ref15] Gong X, Chen W, Gong Y, Zhou L (2006). Clinical analysis of PLI, GI and SBI in patients with fixed orthodontic appliances. Shanghai Kou Qiang Yi Xue.

[ref16] Kaygisiz E, Uzuner FD, Yuksel S, Taner L, Culhaoglu R, Sezgin Y, Ates C (2015). Effects of self-ligating and conventional brackets on halitosis and periodontal conditions. Angle Orthod.

[ref17] Krupińska-Nanys M, Zarzecka J (2015). An assessment of oral hygiene in 7-14-year-old children undergoing orthodontic treatment. J Int Oral Health.

[ref18] Kudirkaite I, Lopatiene K, Zubiene J, Saldunaite K (2016). Age and gender influence on oral hygiene among adolescents with fixed orthodontic appliances. Stomatologija..

[ref19] Kuriakose S, Sundaresan C, Mathai V, Khosla E, Gaffoor FM (2013). A comparative study of salivary buffering capacity, flow rate, resting pH, and salivary Immunoglobulin A in children with rampant caries and caries-resistant children. J Indian Soc Pedod Prev Dent.

[ref20] Lara-Carrillo E, Montiel-Bastida NM, Sánchez-Pérez L, Alanís-Tavira J (2010). Effect of orthodontic treatment on saliva, plaque and the levels of Streptococcus mutans and Lactobacillus. Med Oral Patol Oral Cir Bucal.

[ref21] Levrini L, Mangano A, Montanari P, Margherini S, Caprioglio A, Abbate GM (2015). Periodontal health status in patients treated with the Invisalign® system and fixed orthodontic appliances: A 3-month clinical and microbiological evaluation. Eur J Dent.

[ref22] Li Y, Hu B, Liu Y, Ding G, Zhang C, Wang S (2009). The effects of fixed orthodontic appliances on saliva flow rate and saliva electrolyte concentrations. J Oral Rehabil..

[ref23] Lombardo L, Ortan YÖ, Gorgun Ö, Panza L, Scuzzo G, Siciliani G (2013). Changes in the oral environment after placement of lingual and labial orthodontic appliances. Prog Orthod.

[ref24] Mei L, Chieng J, Wong C, Benic G, Farella M (2017). Factors affecting dental biofilm in patients wearing fixed orthodontic appliances. Prog Orthod.

[ref25] Miethke RR, Vogt S (2007). A comparison of the periodontal health of patients during treatment with the Invisalign system and with fixed orthodontic appliances. J Orofac Orthop.

[ref26] Nalcac R, Ozat Y, Cokakoglu S, Turkkahraman H, Onal S, Kaya S (2014). Effect of bracket type on halitosis, periodontal status, and microbial colonization. Angle Orthod.

[ref27] Naranjo AA, Trivino ML, Jaramillo A, Betancourth M, Botero JE (2006). Changes in the subgingival microbiota and periodontal parameters before and 3 months after bracket placement. Am J Orthod Dentofacial Orthop.

[ref28] Pellegrini P, Sauerwein R, Finlayson T, McLeod J, Covell DA Jr, Maier T (2009). Plaque retention by self-ligating vs elastomeric orthodontic brackets: quantitative comparison of oral bacteria and detection with adenosine triphosphate-driven bioluminescence. Am J Orthod Dentofacial Orthop.

[ref29] Peros K, Mestrovic S, Anic-Milosevic S, Slaj M (2011). Salivary microbial and nonmicrobial parameters in children with fixed orthodontic appliances. Angle Orthod.

[ref30] Petrovic D, Vukic-Culafic B, Ivic S, Djuric M, Milekic B (2013). Study of the risk factors associated with the development of malocclusion. Vojnosanit Pregl.

[ref31] Sánchez ERB, Honores MJC (2015). Effect of orthodontic fixed appliances on salivary flow and viscosity. Rev Mex Ortod.

[ref32] Sanders NL (1999). Evidence-based care in orthodontics and periodontics: a review of the literature. J Am Dent Assoc.

[ref33] Schaefer I, Braumann B (2010). Halitosis, oral health and quality of life during treatment with invisalign and the effect of a lowdose chlorhexidine solution. J Orofac Orthop.

[ref34] Scheerman JF, van Empelen P, van Loveren C, Pakpour AH, van Meijel B, Gholami M, Mierzaie Z, van den Braak MC, Verrips GH (2017). An application of the Health Action Process Approach model to oral hygiene behaviour and dental plaque in adolescents with fixed orthodontic appliances. Int J Pediatr Dent.

[ref35] Silness J, Löe H (1964). Periodontal disease in pregnancy: correlation between oral hygiene and periodontal condition. Acta Odontol Scand.

[ref36] Smiech-Slomkowska G, Jablonska-Zrobek J (2007). The effect of oral health education on dental plaque development and the level of caries-related Streptococcus mutans and Lactobacillus spp. Eur J Orthod.

[ref37] Thie NM, Kato T, Bader G, Montplaisir JY, Lavigne GJ (2002). The significance of saliva during sleep and the relevance of oromotor movements. Sleep Med Rev.

[ref38] Turkkahraman H, Sayin MO, Bozkurt FY, Yetkin Z, Kaya S, Onal S (2005). Archwire ligation techniques, microbial colonization, and periodontal status in orthodontically treated patients. Angle Orthod.

[ref39] Yang X, Su N, Shi Z, Xiang Z, He Y, Han X, Bai D (2017). Effects of self-ligating brackets on oral hygiene and discomfort: a systematic review and meta-analysis of randomized controlled clinical trials. Int J Dent Hyg.

[ref40] Zachrisson BU (1974). Oral hygiene for orthodontic patients: current concepts and practical advice. Am J Orthod Dentofacial Orthop.

